# Extracellular Matrix-Related Gene Signature Implied Immunosuppressive Status and Adverse Survival in Diffuse Glioma Patients

**DOI:** 10.3390/cancers18111720

**Published:** 2026-05-25

**Authors:** Zhuoqun Li, Ping Yang, Zuojun Ning, Ji Shi, Tao Jiang, Jing Chen, Kenan Zhang

**Affiliations:** 1 Department of Molecular Neuropathology, Beijing Neurosurgical Institute, Capital Medical University, Beijing 100070, China; 2 Department of Neurosurgery, Beijing Children’s Hospital, Capital Medical University, National Center for Children’s Health, Beijing 100045, China; 3 Department of Neurosurgery, Beijing Tiantan Hospital, Capital Medical University, Beijing 100070, China

**Keywords:** diffuse glioma, extracellular matrix, tumor microenvironment, prognostic signature

## Abstract

Background: Diffuse glioma is the most common and lethal primary malignant brain tumor, with a complicated tumor microenvironment reported. However, the role of the extracellular matrix (ECM) in diffuse gliomas remains unclear. Methods: To investigate the impact of ECM on glioma malignancy, we constructed an ECM-related signature with six selected prognosis-related genes (VTN, VCAN, TNC, LOXL1, EFEMP1, and AGRN) using the LASSO method. Results: This score was found to significantly differentiate between groups classified by clinical characteristics. Despite elevated immune infiltration and antigen release, the high-ECM-score group exhibited attenuated immune activation with profound exhaustion. Moreover, the ECM score demonstrated accurate prognostic predictive power across three independent validation datasets. Conclusions: Elevated ECM scores characterize an exhausted immune phenotype and are associated with a poorer prognosis in patients with diffuse glioma. This study enhances our understanding of the role of ECM in the malignant progression of gliomas.

## Simple Summary

To study the role of the extracellular matrix (ECM) in diffuse glioma, here, we developed an ECM signature, revealed the correlation between condensed ECM and enriched exhausted immune infiltration, and guided the prognosis assessment of patients.

## 1. Introduction

Diffuse gliomas are the most common primary malignant tumors in the central nervous system (CNS), representing 80% of brain tumors. Treatment regimens have been developed in the past three decades, including maximal tumor resection, radiotherapy, chemotherapy, and physical treatments [1,2]. However, patients still suffer poor survival [3,4]. Highly heterogeneous tumor cells originate from glial or neuronal progenitor cells with diverse oncogenic variations, which induce the failure of targeted therapy [2,5,6].

Recent studies on diffuse glioma have increasingly focused on the tumor microenvironment and cellular interactions [7,8,9]. More and more studies revealed a greatly changed immune infiltration, influencing tumorigenesis, progression, and outcome [10,11,12]. However, the role of the extracellular matrix (ECM) in diffuse glioma remained unclear. ECM is an intricate network of extracellular-secreted macromolecules, including collagens, enzymes, and glycoproteins, mainly dealing with structural scaffolding and biochemical support of cells and tissues [13]. Studies in body cancer revealed that ECM significantly influences tumor proliferation, metastasis, drug resistance, angiogenesis, metabolism, and immunity [14,15,16,17,18,19]. In breast cancer, collagen fibers facilitate tumor cell alignment via DDR1, leading to immune exclusion [20]. All these made it urgent to study the role of ECM in diffuse glioma and develop relevant treatment maneuvers.

In this study, we aimed to explore the critical role of ECM in the development and progression of gliomas. First, we collected 27 ECM-related genes through a literature review and constructed a 6-gene signature using Lasso analysis. Then, we validated the prediction efficiency of the signature in three independent cohorts. Finally, we further explored the biological processes correlated to the ECM score model and validated the different immune statuses in patients.

Our findings provide a powerful prognostic tool for glioma patients, offering new insights into the molecular pathogenesis of glioma and enhancing our understanding of the role of ECM in glioma progression.

## 2. Method

### 2.1. Pathological Slide Collection and Staining

All formalin-fixed paraffin-embedded (FFPE) slides were collected from the Department of Neurosurgery, Beijing Tiantan Hospital, approved by the Ethics Committee of Beijing Tiantan Hospital (Beijing, China), and conducted in accordance with the principles of the Declaration of Helsinki.

Picrosirius red staining was carried out using a Modified Sirius Red Stain Kit (Solarbio, Beijing, China, G1472) following the kit instructions. Briefly, 4 μm FFPE slides were collected, deparaffinized in xylene, and rehydrated through descending concentrations of ethanol to distilled water. Slides were then incubated with freshly prepared iron hematoxylin solution for 10 min and rinsed briefly with distilled water twice. Subsequently, the slides were stained with Sirius red solution for 30 min, followed by rinsing with running water, dehydration in ascending concentrations of ethanol, clearing in xylene, and finally mounted with neutral balsam.

Immunohistochemistry (IHC) staining for LOXL1 (Novus, Centennial, CO, USA, H00004016-D01P, 1:500), Versican (Abcam, Cambridge, UK, ab270445, 1:500), Agrin (Abcam, ab85174, 1:200), Vitronectin (Proteintech, Wuhan, China, 15833-1-AP, 1:200), TNC (Proteintech, Wuhan, China, 67710-1-Ig, 1:500), EFEMP1 (Abcam, Cambridge, UK, ab256457, 1:500), and CD45 (Abcam, Cambridge, UK, ab40763, 1:500) was conducted according to our previous procedures [21].

### 2.2. Datasets Collection

Firstly, the processed transcriptomic expression matrix and corresponding clinical information were obtained from the Chinese Glioma Genome Atlas (CGGA; http://www.cgga.org.cn), including 325 cases from the mRNAseq_325 cohort and 693 cases from the mRNAseq_693 cohort. All patients in the CGGA datasets were Asian patients. The CGGA_325 and CGGA_693 cohorts represent two independent datasets collected at different periods. Then, for external validation, the processed transcriptomic expression matrix of 702 cases was retrieved from The Cancer Genome Atlas (TCGA), along with matched clinical information. The validation cohort was predominantly Caucasian.

Both the cohorts were categorized into five subtypes: lower-grade glioma (LGG), IDH-mutant with 1p/19q codeletion; LGG, IDH-mutant without 1p/19q codeletion; LGG, IDH-wildtype; glioblastoma (GBM), IDH-mutant; and GBM, IDH-wildtype, according to the 2016 WHO classification criteria.

### 2.3. ECM-Related Prognostic Risk Score Model Construction

Univariate Cox proportional hazards regression analysis was first conducted to evaluate the relationship between the expression of ECM-related hub genes and the overall survival (OS) in the CGGA_693 dataset. Subsequently, the least absolute shrinkage and selection operator (LASSO) Cox regression analysis was performed using the R package ‘glmnet’ to reduce dimensionality and identify the most robust prognostic genes. Ten-fold cross-validation was employed to determine the optimal penalty parameter (lambda). To ensure a more parsimonious and stable model while minimizing overfitting, the lambda value corresponding to one standard error from the minimum mean cross-validated error (lambda.1se) was selected as the final threshold. Consequently, 6 key genes (VTN, VCAN, TNC, LOXL1, EFEMP1, and AGRN) were identified from the initial 27 ECM-related candidates to construct the final ECM-related gene scoring system. Finally, a weighted risk score was applied to each sample in three datasets using the following formula.

$$ECMscore=\sum_{i=1}^{n}{expr}_{gene(i)}\times {Coeff}_{gene(i)}$$

### 2.4. Gene Set Functional Enrichment Analysis

Pearson correlation analysis was performed to identify the genes correlated to the ECM score. Genes with a correlation coefficient greater than 0.5 and a *p*-value less than 0.05 were selected for subsequent over-representation analysis (ORA), which was conducted using Metascape (v3.5, http://metascape.org). Additionally, gene set enrichment analysis (GSEA) was carried out using the “enrichplot” R package to identify significantly enriched gene sets, with significance defined by an FDR-adjusted *p*-value < 0.05.

### 2.5. Immune Phenotype Estimation

The ESTIMATE package (version 1.0.13) was first applied to evaluate the immune cell infiltration (immune score) and stromal content (stromal score) for each sample [22]. The CIBERSORT algorithm was then applied to estimate the relative proportions of 22 immune cell types [23].

Tracking Tumor Immunophenotype (TIP) analysis was performed using the online tool in accordance with the protocol provided on the website [24]. The transcriptomic expression matrix was used as input for the analysis. The scores of individual immune cell populations recruited in step 4 were aggregated to obtain the overall score for “Step 4: Trafficking of immune cells to tumor”. An immune exhaustion-related gene set was retrieved from the MSigDB database, and its enrichment score was calculated using single-sample gene set enrichment analysis (ssGSEA) implemented in the “GSVA” R package.

### 2.6. Statistical Analysis

Considering the proximity of the calculated “surv_cutpoint” to the sample median, patients were ultimately dichotomized using the median ECM score. This categorization strategy was adopted to ensure a symmetrical grouping that reflects the central tendency of the ECM score distribution.

All the analyses were conducted in the R software (v4.3) and visualized with the R package “ggplot2” (v3.5.2). A *p*-value less than 0.05 was considered statistically significant.

Student’s *t*-test was applied to evaluate the differences between the two groups. The Kaplan–Meier estimator with the log-rank test was used to determine the survival differences between groups. All analyses were conducted in R software with the packages of “survival” (v3.6-4) and “survminer” (v0.5.0).

The independent prognostic factors were identified using univariate and multivariate Cox regression analyses and visualized with the “forestplot” package (v3.1.7). An individualized prediction model was further developed using the “survival” and “rms” packages. And the predictive performance of the prognostic model was evaluated using receiver operating characteristic (ROC) curves implemented with the “pROC” package (v1.18.5).

## 3. Results

### 3.1. ECM in Diffuse Glioma Cohort

**Figure 1:**
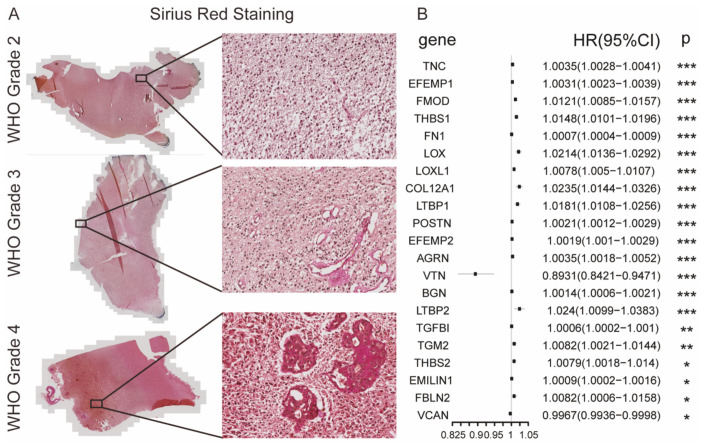
Construction of a prognostic ECM signature for OS by LASSO analysis. (**A**) Collagen content in clinical samples from glioma patients was assessed using Sirius red staining. (**B**) Univariate Cox regression results for the 21 genes in the CGGA dataset. *** *p* < 0.001; ** *p* < 0.01; * *p* < 0.05.

We first performed Picrosirius red staining on clinical samples from diffuse glioma patients with different histological grades. The results revealed a higher content of collagen fibers in high-grade glioma samples compared to lower-grade glioma samples (Figure 1A). To further investigate the impact of the extracellular matrix on malignant progression in gliomas, we reviewed the relevant literature and compiled a list of genes associated with the extracellular matrix. From this list, we identified 21 genes that were statistically related to the overall survival (OS) of glioma patients in the CGGA dataset (Figure 1B). These implied a solid correlation between ECM and the survival of diffuse glioma patients.

### 3.2. Construction of a Prognostic ECM Signature in Glioma

**Figure 2:**
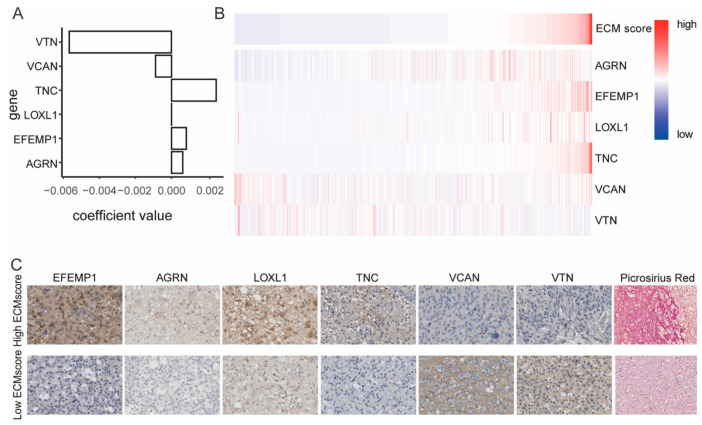
Relationship between the signature genes and ECM score. (**A**) Coefficient values of the six selected genes by LASSO. (**B**) A heatmap of the relationship between the ECM score and six genes. (**C**) VTN, VCAN, TNC, LOXL1, EFEMP1, and AGRN protein expression in the high- and low-ECM groups by IHC staining.

Based on the prognostic roles of the ECM-related genes, we further aimed to construct a prognostic ECM score. We first employed the LASSO regression algorithm to reduce the list of genes and constructed a prognostic ECM signature, including VTN, VCAN, TNC, LOXL1, EFEMP1, and AGRN (Figure 2A). Among these, TNC, LOXL1, EFEMP1, and AGRN positively contributed to the signature scores, whereas VTN and VCAN contributed negatively. The correlation between the selected genes and the ECM signature is shown in a heatmap (Figure 2B). And the expression of the protein level was further validated by IHC staining (Figure 2C). Picrosirius red staining also showed a significant difference between the high- and low-ECM score groups (Figure 2C).

### 3.3. Association of the ECM Score with Clinical Features and Malignant Phenotype in Glioma

**Figure 3:**
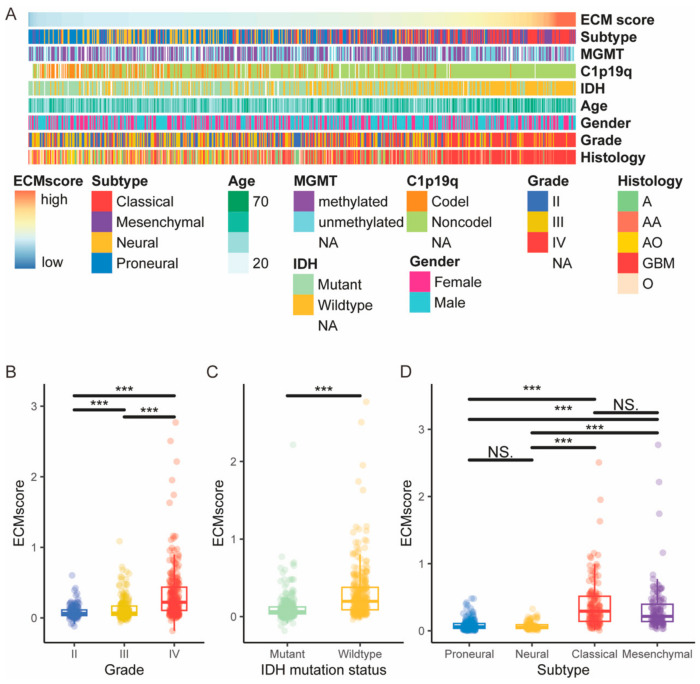
Association of ECM scores with clinicopathological features in the CGGA_693 cohort. (**A**) Landscape of clinical and pathological attributes across patients ranked by ascending ECM scores. (**B**–**D**) Comparison of ECM score levels among different histological grades (**B**), IDH mutation statuses (**C**), and TCGA transcriptomic subtypes (**D**). *** *p* < 0.001; NS. non-significant.

To further study the relationship between the ECM score and clinical characteristics, we arranged the patients based on their ECM score and showed the corresponding pathological characteristics in a heatmap. With an increase in ECM score, higher histological grade, age of diagnosis, and higher proportion of IDH-wildtype and chromosome 1p/19q non-codeletion could be observed (Figure 3A and Figure S1A). Moreover, the ECM score was significantly higher in high WHO grade and IDH wildtype patients in the CGGA dataset (Figure 3B,C). Similar results were validated in a larger sample size in the TCGA dataset (Supplementary Figure S1B,C). Additionally, ECM scores were higher in the classical and mesenchymal subtypes (Figure 3D and Figure S1B,C). Collectively, these findings suggested that the ECM score is closely associated with clinical features and the malignant phenotype of gliomas.

### 3.4. The ECM Score Is Strongly Associated with Immune Functions in Glioma

**Figure 4:**
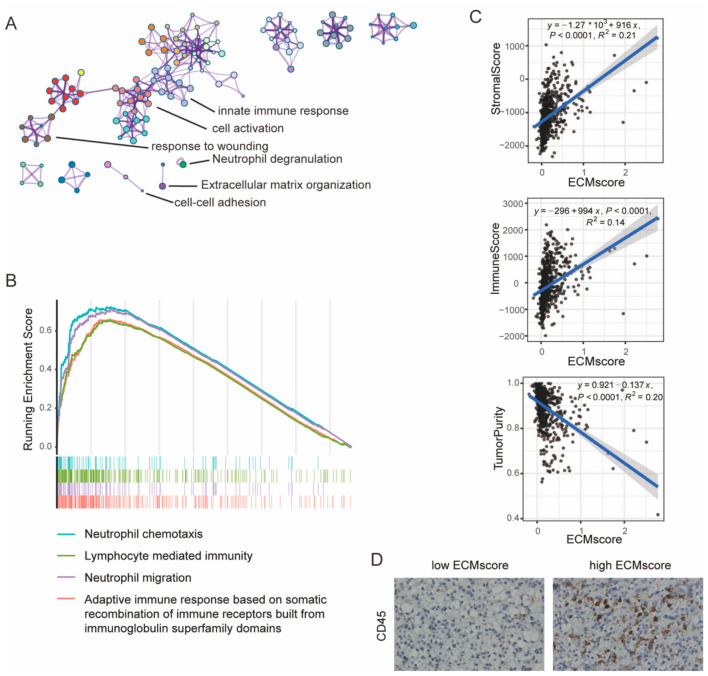
GO and GSEA annotation of genes associated with ECM score in the CGGA_693 dataset. (**A**) Functional enrichment of the positive related genes with the ECM score. (**B**) GSEA results showed enrichment of dampened antitumor immunity in the high ECM score group. (**C**) Scatter plots showed the correlation between stroma score, immune score, tumor purity, and the ECM score. (**D**) IHC staining for CD45 in the high- and low-ECM score samples.

To study the biological principles behind the ECM score, we identified the significantly correlated genes of the score and used the Metascape analysis to identify the enriched gene ontology terms. ECM-correlated genes were significantly enriched in biological processes, including innate immune response, response to wounding, extracellular matrix organization, and neutrophil degranulation (Figure 4A and Figure S2A,B). GSEA analyses further validated that the significantly changed immune-related pathways were enriched in the high-ECM score group in both the CGGA_693 and CGGA_325 datasets. These pathways included adaptive immune response mediated by somatic recombination of immunoglobulin superfamily receptors, lymphocyte-mediated immunity, as well as neutrophil chemotaxis and migration (Figure 4B and Figure S2C).

To further investigate the correlation between the ECM score and the tumor microenvironment (TME), we calculated the correlations between the ECM score and the stromal, tumor purity, and immune scores in the CGGA_693 and CGGA_325 datasets. The results showed that tumors with high ECM scores were found to exhibit higher stromal scores and greater immune cell infiltration but lower tumor purity (Figure 4C and Figure S2D). The increased infiltration of immune cells could be validated by IHC staining of CD45^+^ cells in slides with high ECM scores (Figure 4D). These results indicated that the ECM score can represent increased immune infiltration.

### 3.5. The ECM Score Is Associated with Immune Checkpoint and T-Cell Exhaustion

**Figure 5:**
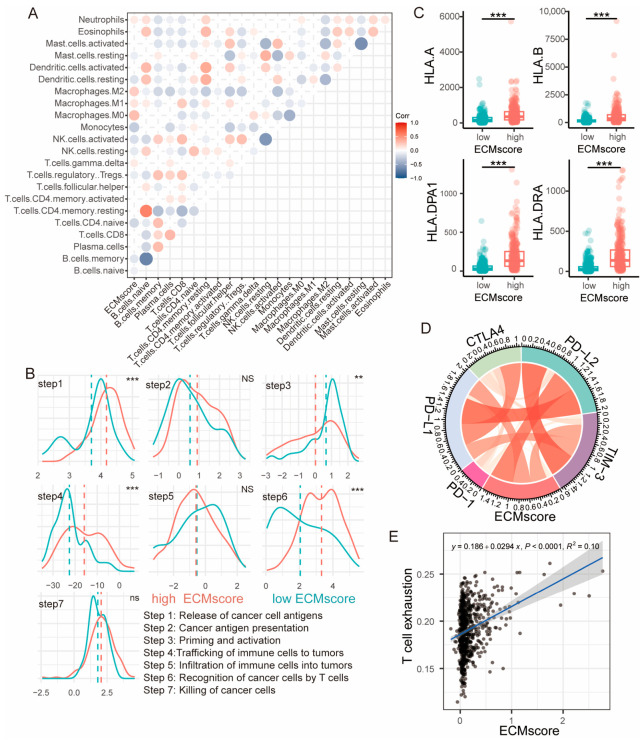
The relationship between ECM score and tumor immune response. (**A**) The correlation coefficient between the ECM score and immune infiltrating cells. (**B**) Differences in various steps of the Cancer-Immunity Cycle in GBM from the CGGA_693 database. (**C**) The association between the ECM score with HLA molecules. (**D**) The correlation coefficient between the ECM score and immune checkpoints. (**E**) The correlation between ECM score and T cell exhaustion index was analyzed by Pearson correlation analysis. *** *p* < 0.001; ** *p* < 0.01; ns. non-significant.

To further study the immune infiltration correlated to the ECM score, we utilized the CIBERSORT algorithm to calculate immune cell infiltration within the tumor microenvironment. Fourteen types of immune cells were identified as correlated with the ECM score. Macrophages and neutrophils exhibited a positive correlation with the ECM score, whereas other cell types, especially the lymphoid cells, showed an inverse association (Figure 5A and Figure S3A).

To further study the detailed immunophenotypes correlated to the ECM score, we applied TIP analysis to analyze the immune infiltration to anti-tumor killing ability and compared them with the ECM score. In both GBM and LGG patients, a higher extracellular matrix (ECM) score was found to be associated with step1: increased release of cancer cell antigens and step4: trafficking of immune cells to the tumor site (Figure 5B, Figures S3B and S4A,B). The increased release of cancer cell antigens was further validated by the higher expression of HLA molecules in the high-ECM score group (Figure 5C and Figure S3C). Notably, despite the facilitated trafficking of immune cells (Step 4) in high-ECM tumors, the actual infiltration (Step 5) and the preceding priming/activation (Step 3) were significantly hindered (Figure 5B, Figures S3B and S4A,B). To further elucidate reduced priming and activation of immune responses in the high-ECM score group, we analyzed the expression levels of immune checkpoints and T-cell exhaustion within the tumors. The results showed that CTLA4, PD-L1, PD-L2, and TIM3 were positively correlated with the ECM score (Figure 5D and Figure S3D). T-cell exhaustion score established by ssGSEA revealed a positive correlation between T-cell exhaustion and the ECM score in the CGGA_693 and CGGA_325 datasets (Figure 5E and Figure S3E).

### 3.6. The ECM Score Served as an Independent Prognostic Factor

**Figure 6:**
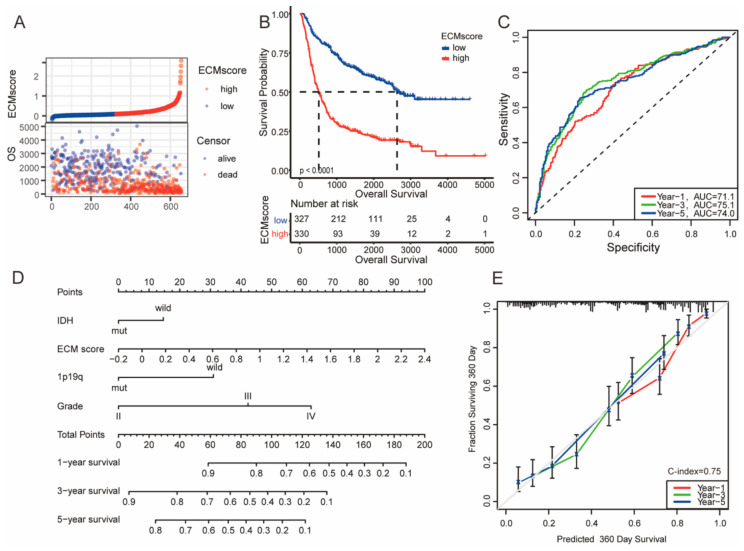
Prognostic utility of the six-gene signature within the CGGA_693 cohort. (**A**) ECM score distribution map illustrating the link between ECM scores (top) and patient survival status (bottom). (**B**) Kaplan–Meier analysis revealing significant survival divergence between ECM-stratified groups. The dashed lines indicate the median overall survival time for each group. (**C**) ROC analysis quantifying the signature’s prognostic accuracy, where the dashed diagonal line represents the reference line (AUC = 0.5). (**D**) Development of a nomogram for clinical outcome forecasting. (**E**) Calibration plots validating the prognostic model. The diagonal gray line represents the ideal reference line; higher overlap between the colored performance lines and the diagonal gray line denotes superior predictive consistency.

Finally, we studied the prognostic role of the ECM score in both the discovery and validation cohorts. Using the median ECM score as a threshold, patients were classified into high- and low-ECM groups. Patients with high ECM scores had a higher mortality rate and shorter overall survival compared to those with low-ECM scores (Figure 6A,B). The prognostic potency of the ECM score appears to be context-dependent. While it effectively predicts shorter survival in IDH-wildtype and IDH-mutant/1p19q-non-codeleted patients, it is not a significant determinant of prognosis for those harboring 1p19q-codeletion (Supplementary Figure S5A–C). The ROC plot illustrated a good performance of the ECM score, with an area under the curve (AUC) of 0.711 at 1 year, 0.751 at 3 years, and 0.740 at 5 years. Similar performance can be validated in the CGGA_325 and TCGA datasets (Supplementary Figure S5D,E).

The ECM score was further identified as an independent factor from other clinical characteristics by the univariate and multivariate Cox regression (Table 1) analyses. A nomogram was further constructed with the independent factors in the Cox regression result. The ECM score contributed significantly to the prediction model, with scores ranging from 0 to 100 (Figure 6D). The concordance index (C-index) for the prediction nomogram was 0.752 in the CGGA_693 dataset, 0.735 in the CGGA_325 dataset, and 0.816 in the TCGA dataset (Figure 6E and Figure S5C,D). The calibration chart demonstrated excellent agreement between predicted and observed 1-year, 3-year, and 5-year OS probabilities in the TCGA and CGGA datasets (Figure 6E and Figure S5F,G), indicating that the signature is highly accurate.

## 4. Discussion

Increasingly, studies have revealed the importance of ECM in cancers, not only influencing the migration and progression of tumor cells but also affecting the infiltration and activation of immune cells, etc. [25]. However, given the distinct immune system in the central nervous system, the role of ECM in diffuse glioma remains unclear. Here, we constructed an ECM-related gene signature in a large-scale diffuse glioma transcriptomic cohort, profiled relevant biological pathways, assessed the immune microenvironment, and finally revealed the independent prognostic role of ECM in diffuse glioma patients.

Some of the selected genes in the signature have been studied in various types of body cancers in recent studies. In breast cancer, TNC, a spatially and temporally restricted extracellular matrix protein, remodeled the extracellular matrix and increased matrix stiffness through the αvβ1/TGF-β signaling pathway [26,27]. The stemness of cancer cells can be enhanced by highly expressed TNC via leucine-rich repeat G protein-coupled receptor 5 (LGR5) and Musashi homolog 1 (MSI1) [28]. In colorectal cancer, AGRN was found to enhance tumor proliferation, migration, and invasion through the Wnt/β-catenin pathway [29,30], and the expression of LOXL1 was positively correlated with epithelial-to-mesenchymal transition (EMT) and increased invasive capabilities [31,32]. In pancreatic carcinoma, EFEMP1 was confirmed to enhance cell growth by binding to EGFR and activating the downstream MAPK/Akt pathway [33]. Interestingly, VTN and VCAN were revealed as oncogenic molecules by various studies but found as protective factors in our study, implying different roles and pathways in the central nervous system [34,35,36,37]. 

To identify the biological pathways related to the ECM score, we enriched the highly expressed genes in high-ECM-score patients and found well-known malignant features [38], including positive regulation of cell motility and the VEGFA-VEGFR2 signaling pathway.

Moreover, the influence of ECM on cancer immunity has been extensively studied in various tumors. We also found various immune response-related terms in the enriched terms. The terms, including lymphocyte immunity, neutrophil chemotaxis, and neutrophil migration, implied the potential correlation between immune infiltration and ECM construction, which was further validated by bulk RNA-seq deconvolution analyses. Higher complexity of immune cells and abundance of macrophages and neutrophils were shown in high-ECM patients. We further validated the immune infiltration via IHC staining. More CD45^+^ cells were found in high-ECM patients. Studies revealed that T-cell migration can be blocked by a stiffer ECM, acting as a physical barrier. In pancreatic ductal adenocarcinoma (PDAC) and lung cancer models, matrix density and architecture directed T cells to the tumor stroma rather than the tumor cell nests due to impaired infiltration ability [39].

Furthermore, Tracking Tumor Immunophenotype analyses revealed not only immune cells’ trafficking into tumors but also higher cancer antigen release found in high-ECM patients, which was consistent with the higher expression levels of HLA molecules. However, even with highly expressed HLA molecules, the antigen presentation process can still be restrained by the complex ECM construction, which is crucial for antigen presentation and immune activation [40]. The complex ECM components can induce the DC phenotype transformation with reduced immunogenicity [41]. DCs cultured on laminins exhibited upregulation of AKT and MEK signaling pathways while showing diminished immunological capacities, which contribute to ovarian cancer progression [42]. Additionally, the rigid ECM can form a barrier around tumor cells, limiting CD8 T-cell recognition, somewhat explaining the difference in antigen release but not differences in cytotoxicity. Dense ECM may facilitate the initial trafficking of immune cells to the tumor site but restricts their effective infiltration into the malignant parenchyma. In this scenario, the ECM-rich stroma may act as a “functional barrier” that limits physical interactions between cytotoxic T cells and tumor cells, thereby facilitating immune evasion and exhaustion. Recent studies also revealed several ECM sensors in infiltrated T cells, which aggravate T-cell exhaustion. The interaction between extracellular collagen and LAIR1 was identified to induce failure of ICI treatment through T-cell exhaustion [43]. Osr2 was also identified as a sensor of stiff ECM in infiltrated T cells in hepatocellular carcinoma [44].

The higher immune checkpoint markers were also found to be highly expressed in high-ECM patients, which explains why the high-ECM scoring group has lower immune priming and activation. Recent studies revealed that higher PD-L1 expression could be induced by a stiffer substrate in lung cancer cells via an Actin-dependent mechanism, aiding tumor immune evasion and growth [40,45]. Further, the T-cell exhaustion was also found to be positively correlated with the ECM score, consistent with the expression level of immune checkpoint markers. Given the immunosuppressive status of the immune response, the antigen presentation failed to elicit an effective immune response in high-ECM patients. Our findings suggest that, although the high-ECM-score subgroup exhibits a suppressed immune state, the concurrent elevation of checkpoint molecules implies that these patients might benefit from immune checkpoint inhibitor (ICI) therapies.

Despite the observed variations in ECM score magnitude between the CGGA and TCGA datasets—likely reflecting ethnic differences—the signature’s predictive power remained stable. Crucially, in all three independent cohorts, elevated ECM scores were invariably linked to diminished overall survival. This indicates that the association between ECM-mediated immune evasion and poor prognosis is a conserved biological phenomenon, independent of the patient’s ethnic background.

Finally, the ECM score demonstrated robust prognostic performance across three independent datasets, which is also consistent in stratified analyses based on WHO grade, IDH mutation status, and chr 1p/19q co-deletion. Multivariate analysis confirmed that the ECM score served as an independent predictor of overall survival after adjusting for potential confounders, highlighting its solid prognostic role across diverse cohorts and subgroups.

Despite the robust prognostic performance of the six-gene signature, we still have to acknowledge the limitation that the underlying molecular mechanisms of each gene in promoting tumor malignancy or immune suppression were not extensively characterized. Consequently, future research will focus on these signature-related genes to systematically investigate their biological contributions to ECM remodeling, immune microenvironment modulation, and the malignant progression of glioma through rigorous in vitro and in vivo experiments.

In summary, we constructed an ECM-related score, which has a favorable efficiency in predicting the prognosis of patients with glioma, and validated the role of ECM in the immune microenvironment of diffuse glioma.

## 5. Conclusions

Here, we demonstrate that elevated ECM scores are strongly associated with poorer prognosis in diffuse glioma. Although higher ECM scores correlate with increased immune infiltration, they are also linked to more exhausted immune cells, indicating a dysfunctional immune microenvironment. These findings suggest that ECM remodeling plays a critical role in shaping tumor–immune interactions and can serve as a valuable prognostic indicator as well as a potential therapeutic target in glioma.

## Figures and Tables

**Table 1:** Univariate and multivariate Cox regression analyses of the association between clinic pathological factors and OS of patients.

Characteristic	Univariate Analysis			Multivariate Analysis		
	HR	95% CI	*p* Value	HR	95% CI	*p* Value
Gender	1.1	0.86–1.4	0.44633	-	-	-
1p/19q status	0.29	0.2–0.42	<0.001	0.57	0.36–0.9	0.01617
IDH status	0.32	0.25–0.41	<0.001	0.73	0.54–0.99	0.04408
Histology	1.16	1.06–1.27	0.00145	0.86	0.7–1.06	0.16347
WHO Grade	2.72	2.26–3.27	<0.001	2.43	1.73–3.41	<0.001
Chemotherapy	1.07	0.8–1.44	0.64294	-	-	-
Radiotherapy	1.2	0.88–1.64	0.24845	-	-	-
MGMT promotor status	0.79	0.62–1.01	0.05492	-	-	-
ECM score	6.3	4.5–8.81	<0.001	2.51	1.58–3.98	<0.001

## Data Availability

The datasets analyzed in this study are publicly available from the Chinese Glioma Genome Atlas (CGGA, http://www.cgga.org.cn/).

## Supplementary Materials

The following supporting information can be downloaded at: https://www.mdpi.com/article/10.3390/cancers18111720/s1, Figure S1: Relationship between the ECM score and the pathological characteristics in the CGGA_325 and TCGA datasets; Figure S2: GO and GSEA annotation of genes associated with the ECM score in the CGGA_325 dataset; Figure S3: The relationship between ECM score and tumor immune response in the CGGA_325 dataset; Figure S4: Differences in various steps of the Cancer-Immunity Cycle in LGG; Figure S5: The prognostic value of the six-gene prognostic signature in the CGGA_325 and TCGA datasets.

## References

[1] van den Bent M.J., Franceschi E., Touat M., French P.J., Idbaih A., Lombardi G., Rudaa R., Schweizer L., Capper D., Sanson M. (2025). Updated EANO guideline on rational molecular testing of gliomas, glioneuronal, and neuronal tumors in adults for targeted therapy selection—Update 1. Neuro Oncol..

[2] Jiang T., Nam D.H., Ram Z., Poo W.S., Wang J., Boldbaatar D., Mao Y., Ma W., Mao Q., You Y. (2026). Updated clinical practice guidelines for the management of adult diffuse gliomas. Cancer Lett..

[3] Ostrom Q.T., Price M., Neff C., Cioffi G., Waite K.A., Kruchko C., Barnholtz-Sloan J.S. (2022). CBTRUS Statistical Report: Primary Brain and Other Central Nervous System Tumors Diagnosed in the United States in 2015–2019. Neuro Oncol..

[4] Zhang K., Liu X., Li G., Chang X., Li S., Chen J., Zhao Z., Wang J., Jiang T., Chai R. (2022). Clinical management and survival outcomes of patients with different molecular subtypes of diffuse gliomas in China (2011–2017): A multicenter retrospective study from CGGA. Cancer Biol. Med..

[5] Verhaak R.G., Hoadley K.A., Purdom E., Wang V., Qi Y., Wilkerson M.D., Miller C.R., Ding L., Golub T., Mesirov J.P. (2010). Integrated genomic analysis identifies clinically relevant subtypes of glioblastoma characterized by abnormalities in PDGFRA, IDH1, EGFR, and NF1. Cancer Cell.

[6] Neftel C., Laffy J., Filbin M.G., Hara T., Shore M.E., Rahme G.J., Richman A.R., Silverbush D., Shaw M.L., Hebert C.M. (2019). An Integrative Model of Cellular States, Plasticity, and Genetics for Glioblastoma. Cell.

[7] Du Y., Shi J., Wang J., Xun Z., Yu Z., Sun H., Bao R., Zheng J., Li Z., Ye Y. (2024). Integration of Pan-Cancer Single-Cell and Spatial Transcriptomics Reveals Stromal Cell Features and Therapeutic Targets in Tumor Microenvironment. Cancer Res..

[8] Li Y., Hu X., Lin R., Zhou G., Zhao L., Zhao D., Zhang Y., Li W., Zhang Y., Ma P. (2022). Single-cell landscape reveals active cell subtypes and their interaction in the tumor microenvironment of gastric cancer. Theranostics.

[9] Cohen M., Giladi A., Barboy O., Hamon P., Li B., Zada M., Gurevich-Shapiro A., Beccaria C.G., David E., Maier B.B. (2022). The interaction of CD4(+) helper T cells with dendritic cells shapes the tumor microenvironment and immune checkpoint blockade response. Nat. Cancer.

[10] Tian J., Cai Y., Li Y., Lu Z., Huang J., Deng Y., Yang N., Wang X., Ying P., Zhang S. (2021). CancerImmunityQTL: A database to systematically evaluate the impact of genetic variants on immune infiltration in human cancer. Nucleic Acids Res..

[11] Virassamy B., Caramia F., Savas P., Sant S., Wang J., Christo S.N., Byrne A., Clarke K., Brown E., Teo Z.L. (2023). Intratumoral CD8(+) T cells with a tissue-resident memory phenotype mediate local immunity and immune checkpoint responses in breast cancer. Cancer Cell.

[12] Anadon C.M., Yu X., Hanggi K., Biswas S., Chaurio R.A., Martin A., Payne K.K., Mandal G., Innamarato P., Harro C.M. (2022). Ovarian cancer immunogenicity is governed by a narrow subset of progenitor tissue-resident memory T cells. Cancer Cell.

[13] He Y., Liu T., Dai S., Xu Z., Wang L., Luo F. (2021). Tumor-Associated Extracellular Matrix: How to Be a Potential Aide to Anti-tumor Immunotherapy?. Front. Cell Dev. Biol..

[14] Alonso-Nocelo M., Raimondo T.M., Vining K.H., Lopez-Lopez R., de la Fuente M., Mooney D.J. (2018). Matrix stiffness and tumor-associated macrophages modulate epithelial to mesenchymal transition of human adenocarcinoma cells. Biofabrication.

[15] Nasrollahi S., Walter C., Loza A.J., Schimizzi G.V., Longmore G.D., Pathak A. (2017). Past matrix stiffness primes epithelial cells and regulates their future collective migration through a mechanical memory. Biomaterials.

[16] Nguyen T.V., Sleiman M., Moriarty T., Herrick W.G., Peyton S.R. (2014). Sorafenib resistance and JNK signaling in carcinoma during extracellular matrix stiffening. Biomaterials.

[17] Bordeleau F., Mason B.N., Lollis E.M., Mazzola M., Zanotelli M.R., Somasegar S., Califano J.P., Montague C., LaValley D.J., Huynh J. (2017). Matrix stiffening promotes a tumor vasculature phenotype. Proc. Natl. Acad. Sci. USA.

[18] Liu H.H., Xu Y., Li C.J., Hsu S.J., Lin X.H., Zhang R., Chen J., Chen J., Gao D.M., Cui J.F. (2022). An SCD1-dependent mechanoresponsive pathway promotes HCC invasion and metastasis through lipid metabolic reprogramming. Mol. Ther..

[19] Kuczek D.E., Larsen A.M.H., Thorseth M.L., Carretta M., Kalvisa A., Siersbaek M.S., Simoes A.M.C., Roslind A., Engelholm L.H., Noessner E. (2019). Collagen density regulates the activity of tumor-infiltrating T cells. J. Immunother. Cancer.

[20] Su H., Yang F., Fu R., Trinh B., Sun N., Liu J., Kumar A., Baglieri J., Siruno J., Le M. (2022). Collagenolysis-dependent DDR1 signalling dictates pancreatic cancer outcome. Nature.

[21] Ma W., Zhang K., Bao Z., Jiang T., Zhang Y. (2021). SAMD9 Is Relating With M2 Macrophage and Remarkable Malignancy Characters in Low-Grade Glioma. Front. Immunol..

[22] Yoshihara K., Shahmoradgoli M., Martinez E., Vegesna R., Kim H., Torres-Garcia W., Trevino V., Shen H., Laird P.W., Levine D.A. (2013). Inferring tumour purity and stromal and immune cell admixture from expression data. Nat. Commun..

[23] Newman A.M., Liu C.L., Green M.R., Gentles A.J., Feng W., Xu Y., Hoang C.D., Diehn M., Alizadeh A.A. (2015). Robust enumeration of cell subsets from tissue expression profiles. Nat. Methods.

[24] Xu L., Deng C., Pang B., Zhang X., Liu W., Liao G., Yuan H., Cheng P., Li F., Long Z. (2018). TIP: A Web Server for Resolving Tumor Immunophenotype Profiling. Cancer Res..

[25] Wei Y., Chen D., Zhang Q., You F., Fu Y., Zheng L., Zhang L., Zhang N., Liang G., Yang J. (2025). ECM-based molecular subtypes define prognostic, EMT status, and therapeutic diversity in IDH-mutant gliomas. NPJ Precis. Oncol..

[26] Katoh D., Kozuka Y., Noro A., Ogawa T., Imanaka-Yoshida K., Yoshida T. (2020). Tenascin-C Induces Phenotypic Changes in Fibroblasts to Myofibroblasts with High Contractility through the Integrin alphavbeta1/Transforming Growth Factor beta/SMAD Signaling Axis in Human Breast Cancer. Am. J. Pathol..

[27] Huang Y., Sun M., Lu Z., Zhong Q., Tan M., Wei Q., Zheng L. (2024). Role of integrin beta1 and tenascin C mediate TGF-SMAD2/3 signaling in chondrogenic differentiation of BMSCs induced by type I collagen hydrogel. Regen. Biomater..

[28] Oskarsson T., Acharyya S., Zhang X.H., Vanharanta S., Tavazoie S.F., Morris P.G., Downey R.J., Manova-Todorova K., Brogi E., Massague J. (2011). Breast cancer cells produce tenascin C as a metastatic niche component to colonize the lungs. Nat. Med..

[29] Li X., Lu Y., Wen P., Yuan Y., Xiao Z., Shi H., Feng E. (2023). Matrine restrains the development of colorectal cancer through regulating the AGRN/Wnt/beta-catenin pathway. Environ. Toxicol..

[30] Wang Z.Q., Sun X.L., Wang Y.L., Miao Y.L. (2021). Agrin promotes the proliferation, invasion and migration of rectal cancer cells via the WNT signaling pathway to contribute to rectal cancer progression. J. Recept. Signal Transduct. Res..

[31] Li C., Chen S., Fang X., Du Y., Guan X.Y., Lin R., Xu L., Lan P., Yan Q. (2024). LOXL1 promotes tumor cell malignancy and restricts CD8 + T cell infiltration in colorectal cancer. Cell Biol. Toxicol..

[32] Hu Q., Masuda T., Kuramitsu S., Tobo T., Sato K., Kidogami S., Nambara S., Ueda M., Tsuruda Y., Kuroda Y. (2020). Potential association of LOXL1 with peritoneal dissemination in gastric cancer possibly via promotion of EMT. PLoS ONE.

[33] Camaj P., Seeliger H., Ischenko I., Krebs S., Blum H., De Toni E.N., Faktorova D., Jauch K.W., Bruns C.J. (2009). EFEMP1 binds the EGF receptor and activates MAPK and Akt pathways in pancreatic carcinoma cells. Biol. Chem..

[34] Pola C., Formenti S.C., Schneider R.J. (2013). Vitronectin-alphavbeta3 integrin engagement directs hypoxia-resistant mTOR activity and sustained protein synthesis linked to invasion by breast cancer cells. Cancer Res..

[35] Uhm J.H., Dooley N.P., Kyritsis A.P., Rao J.S., Gladson C.L. (1999). Vitronectin, a glioma-derived extracellular matrix protein, protects tumor cells from apoptotic death. Clin. Cancer Res..

[36] Islam S., Jahan N., Shahida A., Karnan S., Watanabe H. (2022). Accumulation of versican and lack of versikine ameliorate acute colitis. Matrix Biol..

[37] Ping Q., Wang C., Cheng X., Zhong Y., Yan R., Yang M., Shi Y., Li X., Li X., Huang W. (2023). TGF-beta1 dominates stromal fibroblast-mediated EMT via the FAP/VCAN axis in bladder cancer cells. J. Transl. Med..

[38] Pickup M.W., Mouw J.K., Weaver V.M. (2014). The extracellular matrix modulates the hallmarks of cancer. EMBO Rep..

[39] Salmon H., Franciszkiewicz K., Damotte D., Dieu-Nosjean M.C., Validire P., Trautmann A., Mami-Chouaib F., Donnadieu E. (2012). Matrix architecture defines the preferential localization and migration of T cells into the stroma of human lung tumors. J. Clin. Investig..

[40] Blander J.M., Longman R.S., Iliev I.D., Sonnenberg G.F., Artis D. (2017). Regulation of inflammation by microbiota interactions with the host. Nat. Immunol..

[41] Sprague L., Muccioli M., Pate M., Meles E., McGinty J., Nandigam H., Venkatesh A.K., Gu M.Y., Mansfield K., Rutowski A. (2011). The interplay between surfaces and soluble factors define the immunologic and angiogenic properties of myeloid dendritic cells. BMC Immunol..

[42] Phillippi B., Singh M., Loftus T., Smith H., Muccioli M., Wright J., Pate M., Benencia F. (2020). Effect of laminin environments and tumor factors on the biology of myeloid dendritic cells. Immunobiology.

[43] Peng D.H., Rodriguez B.L., Diao L., Chen L., Wang J., Byers L.A., Wei Y., Chapman H.A., Yamauchi M., Behrens C. (2020). Collagen promotes anti-PD-1/PD-L1 resistance in cancer through LAIR1-dependent CD8(+) T cell exhaustion. Nat. Commun..

[44] Zhang J., Li J., Hou Y., Lin Y., Zhao H., Shi Y., Chen K., Nian C., Tang J., Pan L. (2024). Osr2 functions as a biomechanical checkpoint to aggravate CD8(+) T cell exhaustion in tumor. Cell.

[45] Shen Y., Li Y., Zhu Q., Wang J., Huang Y., Liang J., Wu X., Zhao Y. (2022). The immunomodulatory effect of microglia on ECM neuroinflammation via the PD-1/PD-L1 pathway. CNS Neurosci. Ther..

