# Supplementary material for: Extracellular Matrix-Related Gene Signature Implied Immunosuppressive Status and Adverse Survival in Diffuse Glioma Patients
Source: Cancers (Basel). 2026 May 25;18(11):1720. doi: 10.3390/cancers18111720 (PMC13255635; doi:10.3390/cancers18111720)
Supplement: Supplementary file 1 [file cancers-18-01720-s001.zip › cancers-4265399-supplementary.pdf]

cancers

Supplemental Materials

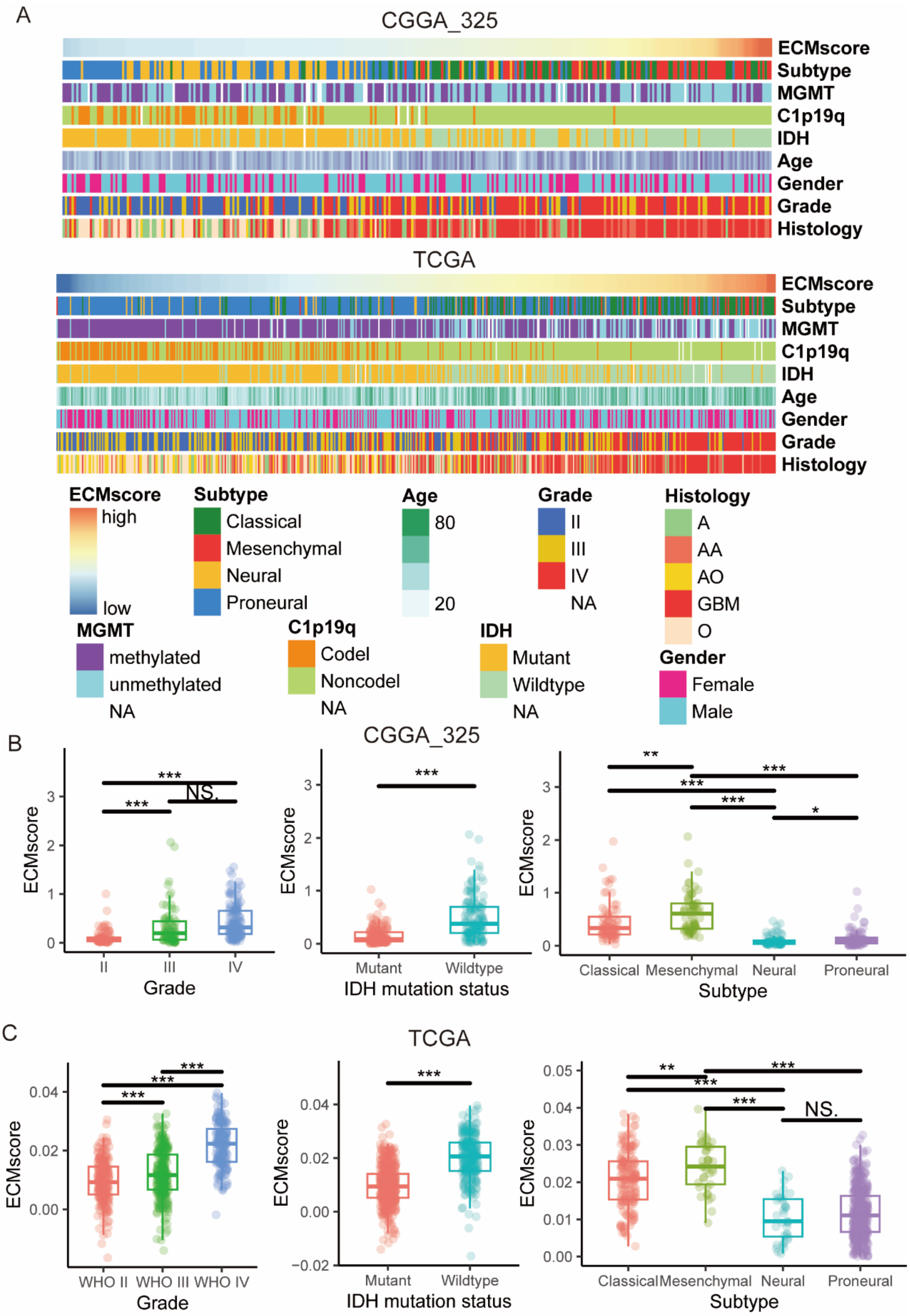

Figure S1. Relationship between the ECM score and the pathological characteristics in the CGGA_325 and TCGA datasets.A) The distribution of clinical and pathological characteristics arranged by the increasing ECM score in the CGGA_325 and TCGA datasets. B) Distribution of ECM score in patients stratified by TCGA subtype, grade, and IDH wildtype status in the CGGA_325 dataset. C) Distribution of ECM score in patients stratified by TCGA subtype, grade, and IDH wildtype status in the TCGA dataset. *** p < 0.001; ** p < 0.01; * p < 0.05; NS. non-significant.

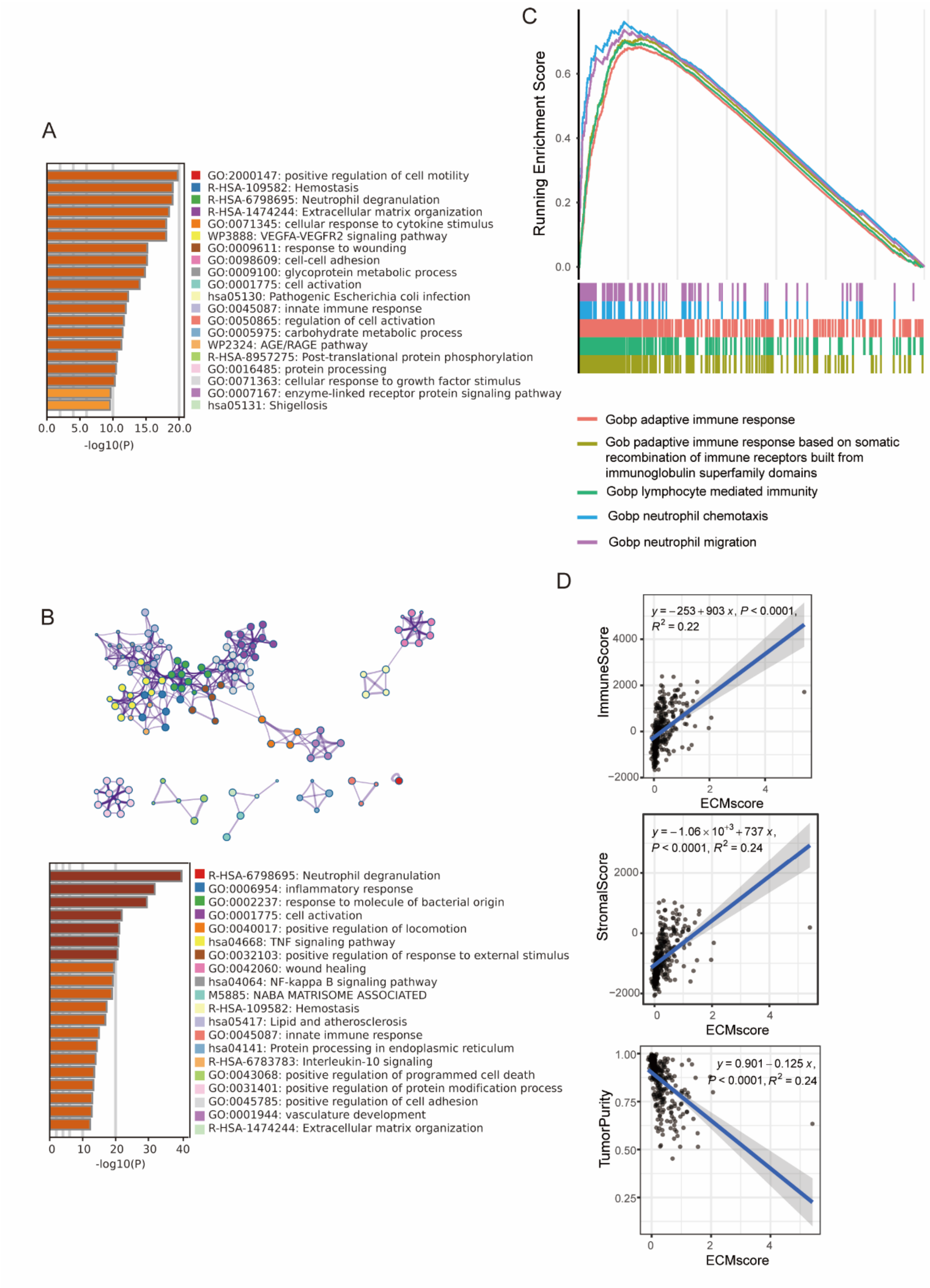

Figure S2. GO and GSEA annotation of genes associated with the ECM score in the CGGA_325 dataset. A) Functional enrichment of the positive related genes with the ECM score by Metascape in the CGGA_693 dataset. B) Functional enrichment of the positive related genes with the ECM score by Metascape in the CGGA_325 dataset. C) GSEA results showed enrichment of dampened anti-tumor immunity in the high ECM score group in CGGA_325 dataset. D) Scatter plots showed the relationship between stroma score, immune score, or tumorpurity and ECM score in CGGA_325 dataset.

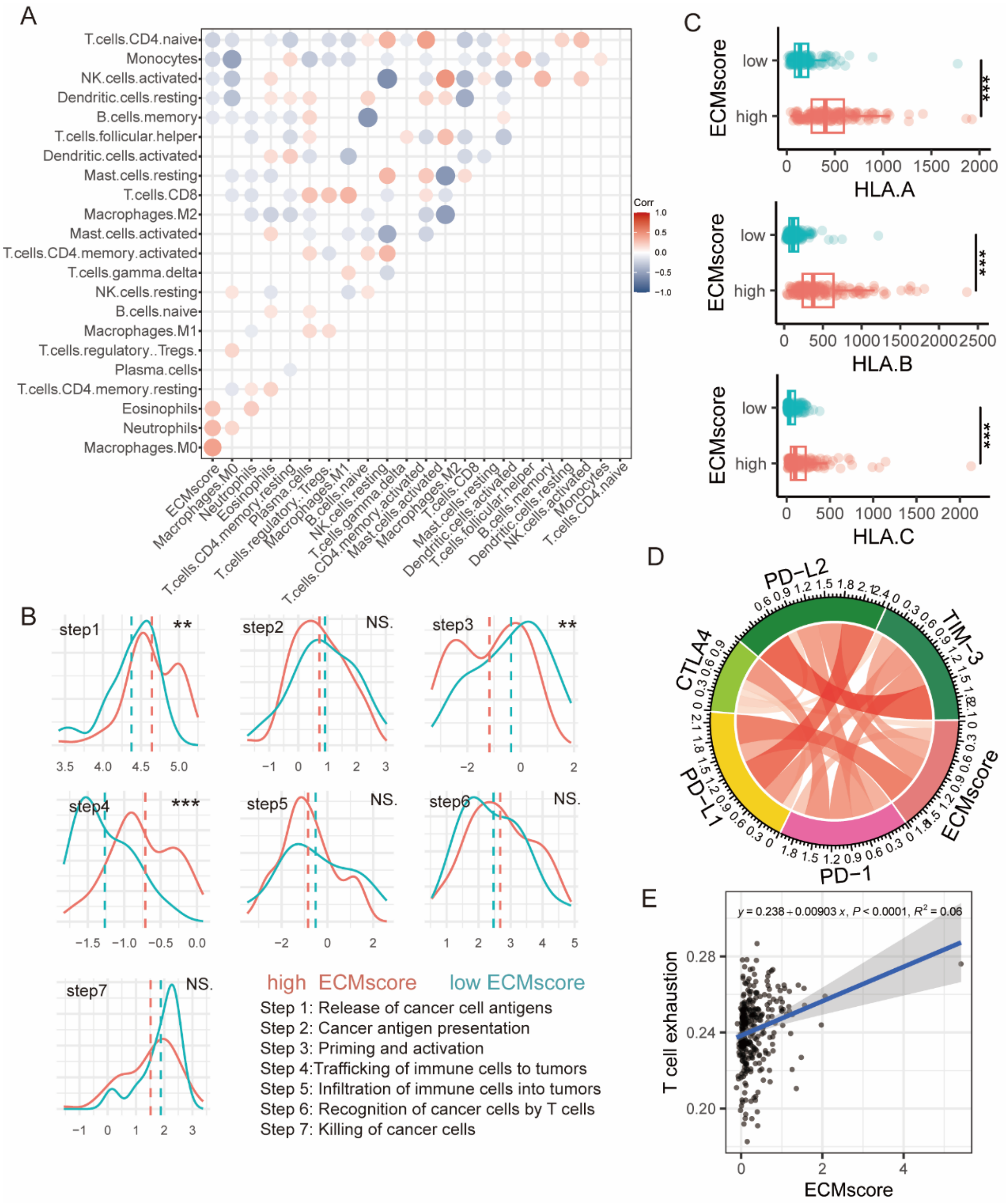

Figure S3. The relationship between ECM score and tumor immune response in the CGGA_325 dataset. A) The correlation coefficient between the ECM score and immune infiltrating cells in the CGGA_325 dataset. B) Differences in various steps of the Cancer-Immunity Cycle in GBM from the CGGA_325 database C) The association between the ECM score with HLA in the CGGA_325 dataset. D) The correlation coefficient between the ECM score and immune checkpoints in the CGGA_325 dataset. E) The correlation between ECM score and T cell exhaustion index was analyzed by Pearson correlation analysis in the CGGA_325 dataset. *** p < 0.001; ** p < 0.01; * p < 0.05; NS. non-significant.

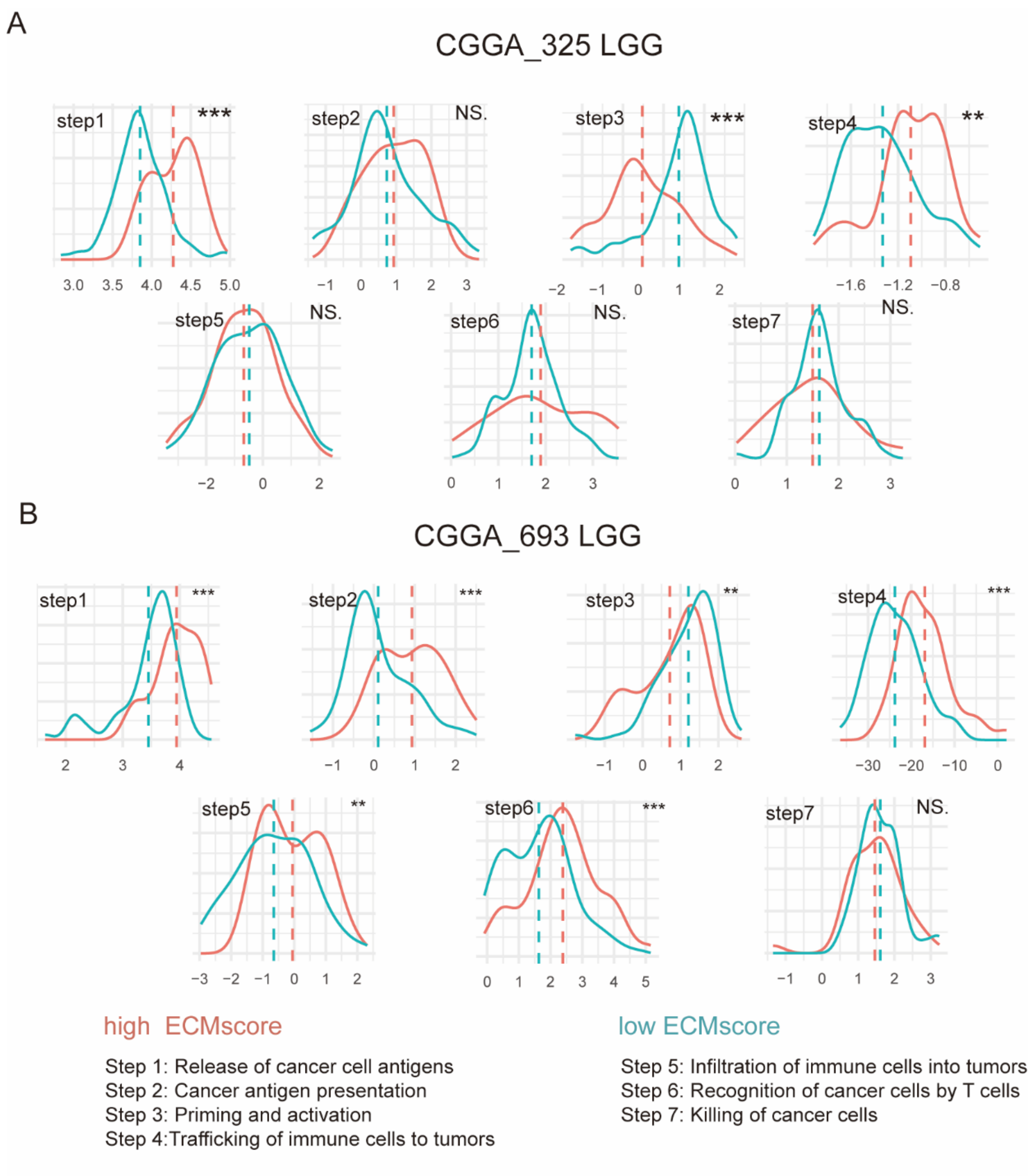

Figure S4. Differences in various steps of the Cancer-Immunity Cycle in LGG. A) Differences in various steps of the Cancer-Immunity Cycle in LGG from the CGGA_325 database B) Differences in various steps of the Cancer-Immunity Cycle in LGG from the CGGA_693 database *** $p < 0.001$; ** $p < 0.01$; * $p < 0.05$; NS. non-significant.

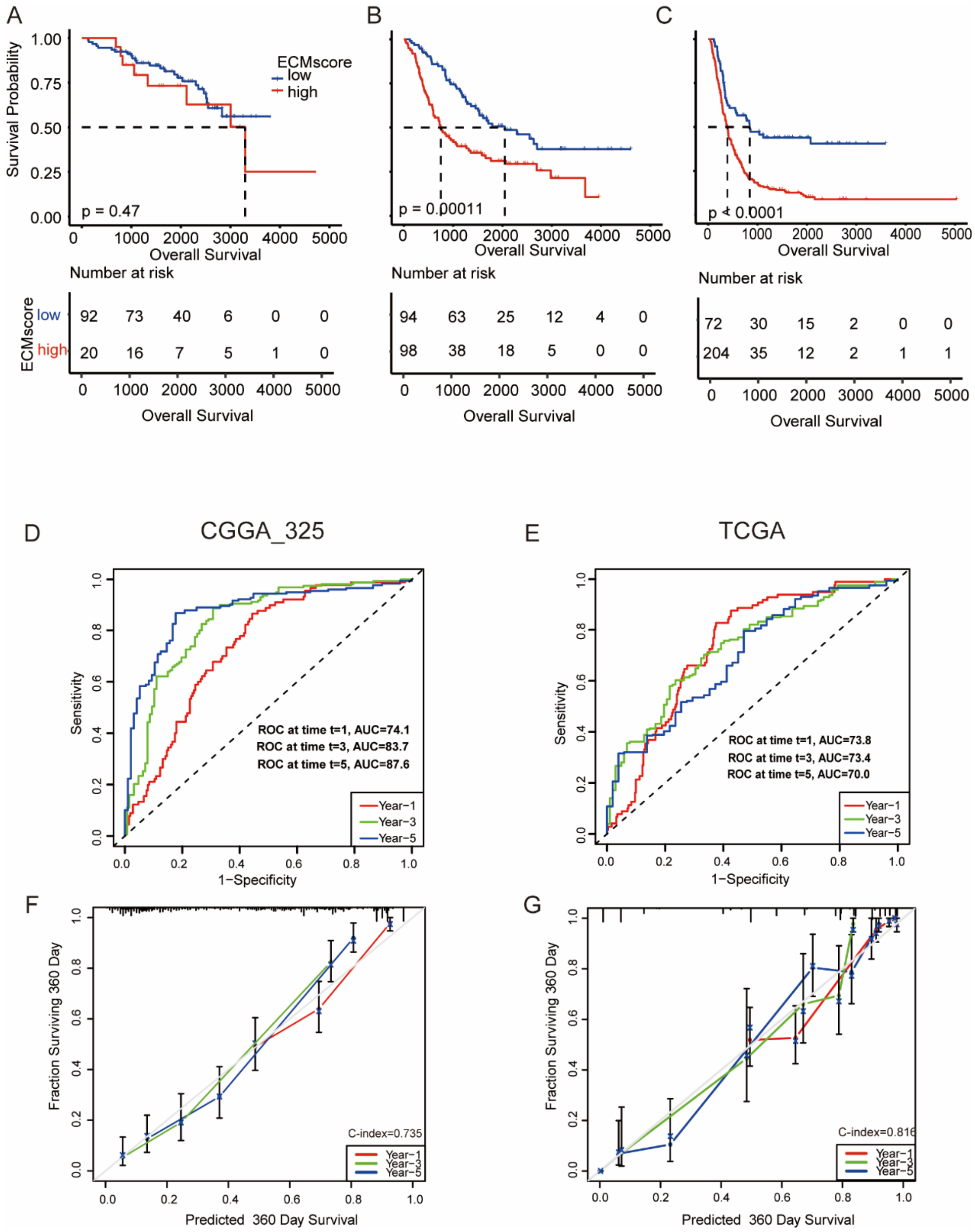

Figure S5. The prognostic value of the six-gene prognostic signature in the CGGA_325 and TCGA datasets. A-C) Kaplan–Meier analysis revealing significant survival divergence between ECM-stratified groups in IDH-mutant and 1p19q-non-codeleted lower-grade glioma subgroup (A), IDH-mutant and 1p19q-non-codeleted lower-grade glioma subgroup(B), and IDH-wildtype GBM subgroup (C). D) ROC curves verified the prognostic performance of the ECM score in the CGGA_325 dataset. E) ROC curves verified the prognostic performance of the ECM score in the TCGA dataset. F) The calibration curve for the nomogram model in the CGGA_325 dataset. G) The calibration curve for the nomogram model in the TCGA dataset.
